# Impact of CRRT in Patients with PARDS Treated with VV-ECMO

**DOI:** 10.3390/membranes11030195

**Published:** 2021-03-11

**Authors:** Sébastien Redant, Océane Barbance, Ashita Tolwani, Xavier Beretta-Piccoli, Jacques Massaut, David De Bels, Fabio S. Taccone, Patrick M. Honoré, Dominique Biarent

**Affiliations:** 1 Departments of Intensive Care, Brugmann University Hospital, 1020 Brussels, Belgium; oceane.barbance@huderf.be (O.B.); Jacques.massaut@chu-brugmann.be (J.M.); david.debels@chu-brugmann.be (D.D.B.); Patrick.Honore@CHU-Brugmann.be (P.M.H.); 2 Departments of Intensive Care, Hospital Universitaire des Enfants Reine Fabiola (HUDERF), 1020 Brussels, Belgium; xavier.beretta-piccoli@huderf.be (X.B.-P.); dominique.biarent@huderf.be (D.B.); 3 Division of Nephrology, University of Alabama at Birmingham School of Medicine, Birmingham, AL 35233, USA; atolwani@uabmc.edu; 4 Department of Intensive Care, Hopital Erasme, Université Libre de Bruxelles, 1020 Brussels, Belgium; Fabio.Taccone@erasme.ulb.ac.be

**Keywords:** ECMO, ARDS, CRRT, AKI, pediatric

## Abstract

The high mortality of pediatric acute respiratory distress syndrome (PARDS) is partly related to fluid overload. Extracorporeal membrane oxygenation (ECMO) is used to treat pediatric patients with severe PARDS, but can result in acute kidney injury (AKI) and worsening fluid overload. The objective of this study was to determine whether the addition of CRRT to ECMO in patients with PARDS is associated with increased mortality. Methods: We conducted a retrospective 7-year study of patients with PARDS requiring ECMO and divided them into those requiring CRRT and those not requiring CRRT. We calculated severity of illness scores, the amount of blood products administered to both groups, and determined the impact of CRRT on mortality and morbidity. Results: We found no significant difference in severity of illness scores except the vasoactive inotropic score (VIS, 45 ± 71 vs. 139 ± 251, *p* = 0.042), which was significantly elevated during the initiation and the first three days of ECMO. CRRT was associated with an increase in the use of blood products and noradrenaline (*p* < 0.01) without changing ECMO duration, length of PICU stay or mortality. Conclusion: The addition of CRRT to ECMO is associated with a greater consumption of blood products but no increase in mortality.

## 1. Introduction

**Figure 1:**
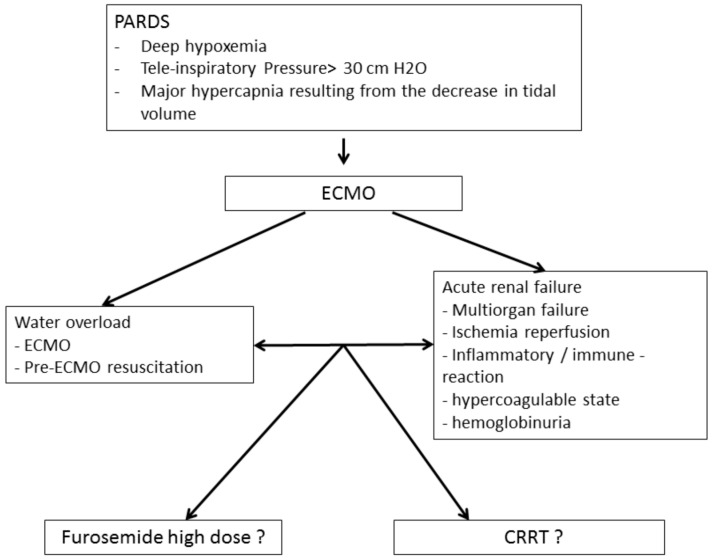
Principle of setting up an ECMO coupled with extra-renal purification.

Pediatric acute respiratory distress syndrome (PARDS) is a rare condition with an incidence that varies from 2.0 to 12.8 per 100,000 children (aged 0 to 15 years) per year [1], representing approximately 3% of patients admitted to the pediatric intensive care unit (PICU) [2]. Although better diagnosis and management have led to improved outcomes in recent years, PARDS is still associated with a mortality ranging between 22 and 40% [3]. Patients with severe forms of PARDS may require veno-venous extracorporeal membrane oxygenation (VV-ECMO). ECMO can lead to worsening fluid overload as a result of resuscitation maneuvers and/or acute kidney injury (AKI), which delays or prevents the restoration of euvolemia (Figure 1).

### 1.1. Fluid Balance in PARDS

Fluid balance is very important in the management of children with PARDS. Fluid overload is associated with increased mortality, decreased oxygenation, prolonged mechanical ventilation time and length of ICU stay [4]. An increase in fluid balance of 10 mL/kg/24 h in patients with acute pulmonary disease has been shown to be associated with an increase in mortality and the duration of mechanical ventilation [5]. A positive fluid balance at day 3 of ICU admission has been shown to be associated with a longer duration of ventilation [6] but with no influence on mortality.

### 1.2. Fluid Overload in ECMO

A weight increase of 5 to 30% is observed when newborns are placed on ECMO [7]. This increase is related to both the fluid required for resuscitation and the effect of ECMO itself. Radioisotope studies have shown an increase in extracellular fluid following initiation of ECMO with a concomitant increase in total body fluid. This phenomenon is due to a significant capillary leak related to hemodynamic instability, hypoxia and acidosis [7]. Fluid overload increases mortality in both ECMO and PARDS [8,9], with a 3% increase in mortality for each percent increase in fluid overload [10]. Several studies have demonstrated a positive fluid balance at ECMO day 3 as an independent predictor of mortality [4,11]. As a result, the guidelines of the Extracorporeal Life Support Organization (ELSO) recommend that the extra-cellular fluid volume be reduced to the patient’s dry weight and maintained [12].

### 1.3. AKI in VV-ECMO

By improving systemic oxygenation, decreasing oxygen uptake and improving hemodynamics, VV-ECMO can improve renal metabolism [13]. However, AKI remains very common (>80%) in PICU patients treated with ECMO for four main reasons. First, the introduction of ECMO can require rapid adjustments of inotropes and vasopressors causing immediate changes in renal perfusion with resultant ischemia and reperfusion injury [13]. Second, inflammation is precipitated by the exposure of blood to an artificial membrane. Furthermore, due to an increase in intestinal permeability, there is an elevation in the levels of both lipopolysaccharide (LPS) [14] and tumor necrosis factor (TNF) levels disproportionate to the presence of receptors at the pulmonary level [15]. Third, a consumptive coagulopathy, especially in the first 24 h of ECMO [16], occurs in addition to hemorrhage and thromboses [17]. Finally, the development of hemolysis with hemoglobinuria is exacerbated by air/fluid contact and the generation of negative pressures [13] when CRRT is connected to the ECMO circuit. The extreme pressure difference between ECMO and CRRT causes a shearing phenomenon that further aggravates hemoglobinuria [18]. Hemoglobinuria jeopardizes not only renal function but also the overall prognosis of the patient [19]. AKI in PARDS is associated with a higher mortality due to fluid overload of the interstitium, decreased oxygen transfer to tissues, and increased extravascular pulmonary fluid with a resultant decrease in oxygen diffusion from the alveoli to the vessels [20].

### 1.4. Indications for CRRT in VV-ECMO

CRRT has become the modality of choice for treating AKI in the intensive care unit due to its beneficial effects on hemodynamics and fluid balance. Indications for the use of CRRT in ECMO patients do not differ from the classic indications for AKI: uremia, acidosis, electrolyte disorders and fluid overload. An international survey of 65 ECMO centers found that the main indications were fluid overload (43%), AKI (35%), fluid overload prevention (16%) and electrolyte disturbances (4%) [21].

### 1.5. Illustrative Case

A 4-month-old infant with RSV pneumonia was admitted to our pediatric intensive care unit for hypoxia. A diagnosis of PARDS was made. He was intubated and protectively ventilated at 6 mL/kg of tidal volume. He gradually deteriorated his pulmonary status until day 10, when he faced respiratory acidosis (pH 7.22) and severe hypoxia (PaO_2_/FiO_2_ ratio 48). The child was placed on VV-ECMO. He unfortunately died 30 h later from cardiac arrest. The cumulative water balance during his stay in the PICU was 346 mL/Kg due to several volume loadings due to hemodynamic instability (VIS score 13). The patient’s renal function was characterized by maximal creatinine of 0.4 mg/dL and minimal creatinine clearance of 69 mL/min. Treatment with furosemide was attempted without recourse to CRRT which could have allowed a greater ultrafiltration and a more effective reduction of the amount of water present in the lungs due to PARDS.

### 1.6. Rationale and Purpose of the Study

In pediatric critically ill patients, the fear of increased mortality has prevented the systematic use of CRRT for fluid overload and instead there is a preference for using high doses of diuretics up to 2 mg/kg/h in addition to fluid restriction (Figure 1). The use of CRRT with ECMO has been shown to not increase the risk of mortality in adults [22]; however, its use in pediatric patients remains controversial, especially in pediatric resuscitation. The primary objective of this study was to investigate whether the addition of CRRT to ECMO in PARDS patients is associated with increased mortality. The secondary objectives were to determine the impact of the addition of CRRT on the use of blood products and the duration of ECMO, mechanical ventilation and PICU stay.

## 2. Methods

### 2.1. Data Collection

We conducted a retrospective study by collecting data from the intensive care management program of Queen Fabiola Children’s University Hospital (HUDERF) on patients with PARDS who received VV-ECMO with and without CRRT. We analyzed data from patients admitted between 1 June 2012 and 31 August 2019. This period corresponded to the installation and use of the Intellispace Critical Care and Anesthesia information system (ICCA; Phillips, Eindhoven, The Netherlands) in our ICU. Demographic data, the diagnosis associated with PARDS, duration of ECMO and CRRT, the delay between ECMO and CRRT, and the quantity of blood derivatives prescribed during the 7 days of ECMO and during the CRRT period were collected. We estimated the average time for setting up CRRT and from this we defined a period of control measurement in the population without CRRT. We also collected data regarding the pH at initiation of ECMO, ventilator settings and the daily vasoactive inotropic score (VIS). The Pediatric Index of Mortality (PIM), Pediatric Risk of Mortality (PRIM), Pediatric Logistic Organ Dysfunction (PELOD) and Pediatric Pulmonary Rescue with ECMO Prediction (P-PREP) scores were calculated.

### 2.2. Definition of Severity of Illness Scores

The Pediatric Pulmonary Rescue with ECMO Prediction (P-PREP) is a score developed and validated with data from the registry of the Extracorporeal Life Support Organization (ESLO), which gathers data from 449 international centers [23]. This score uses pre-ECMO data to predict the in-hospital mortality of children with respiratory failure requiring ECMO. Ninety-five percent of patients from the registry were found to have a score between −1 and +20, while 45% had a score between +3 and +8. Using this scoring system, all patients who had a score ≤ −10 survived (*n* = 15), while patients with a score ≥ 42 died (*n* = 2) [23].

The vasoactive inotropic score (VIS) is calculated as follows: dopamine + dobutamine + 10 × milrinone + 100 × adrenaline + 100 × noradrenaline (all expressed in μg/kg/min) + 10,000 × vasopressin (U/kg/min). This quantifies the amount of inotropes and vasopressors administered, allowing comparison of patients in the context of clinical research.

### 2.3. Definition of AKI

AKI was defined according to the Kidney Disease: Improving Global Outcomes (KDIGO) criteria [24], including a decrease in urine output to <0.5 mL/kg/h over a period of 6 to 12 h and/or an increase in serum creatinine (sCr) > 0.3 mg/dL, or 1.5 times the base value (defined as the lowest creatinine of the stay prior to CRRT). The presence of AKI was assessed over a period of 7 days after the initiation of ECMO. We defined two patient populations: patients requiring CRRT and patients not requiring CRRT.

### 2.4. ECMO and CRRT Technique

Patients were placed on VV-ECMO via a dual-channel Avalon cannula inserted either surgically or percutaneously. Some patients were treated with CRRT while others were treated with fluid restriction and diuretics. The main indication for CRRT was a positive fluid balance despite furosemide at 1 mg/kg/h. CRRT was performed in parallel with the ECMO circuit. The CRRT circuit was placed in the ECMO circuit after the pump while the blood returned from CRRT before the pump, with the advantage of reinjection before the oxygenator to avoid the risk of air embolism. The CRRT mode was continuous veno-venous hemodiafiltration (CVVHDF) using the Prismaflex^®^ system (Baxter International Corporation, Deerfield, Illinois, USA). The ECMO machines used were Maquet consoles (Maquet Cardiopulmonary GmbH, Rastatt, Germany) until December 2017 and then MEDOS Deltastream consoles (XENIOS AG, Heilbronn, Germany).

### 2.5. Endpoints

We measured cumulative and daily fluid balance during a period of 7 days following the initiation of ECMO. We also measured duration of ECMO, mechanical ventilation, and the survival rate.

### 2.6. Statistical Analysis

The data was analyzed via IBM SPSS 23 for Windows with a significance level of *p* < 0.05. The normality of the distribution was tested by a Kolmogorov-Smirnov test. The tests performed on non-normal variables were non-parametric Mann-Whitney tests. The tests on dichotomous variables were chi-squares.

We used a “propensity score” to match each child treated with CRRT to two children who did not have CRRT and whose characteristics were closest as defined by this score. To calculate this score, we determined the variables that were related to treatment or outcome (here death). To do this, we performed two logistic regressions, one for the variables related to treatment, the other for the variables related to death. For treatment (CRRT), maximum creatinine was retained as an independent variable; for death, sepsis, and second probability of death in order of variables in the data table. We used these variables to calculate the “propensity score” using the R software and the “Optmatch” package. Children who have been treated with CRRT can thus each be paired with two control children (no CRRT) with the same “propensity score”.

## 3. Results

We included data from 45 patients undergoing VV-ECMO for severe PARDS (pneumonia: *n* = 22, sepsis *n* = 6 (including 1 with pneumonia), inhalation: *n* = 17). Patient characteristics are shown in Table 1.

The average age was 19 ± 41 months. Sixteen patients were less than 1 month old. All patients were admitted for PARDS of medical origin. The average duration of ECMO was 152 ± 122 h. CRRT was initiated 20 ± 39 h after the start of ECMO. The average P-PREP was 6 ± 4. Of the 45 patients, 40 (88%) developed AKI and 8 required CRRT. The number of patients with KDIGO AKI stage 1, 2 and 3 were 6, 19 and 15, respectively.

**Figure 2:**
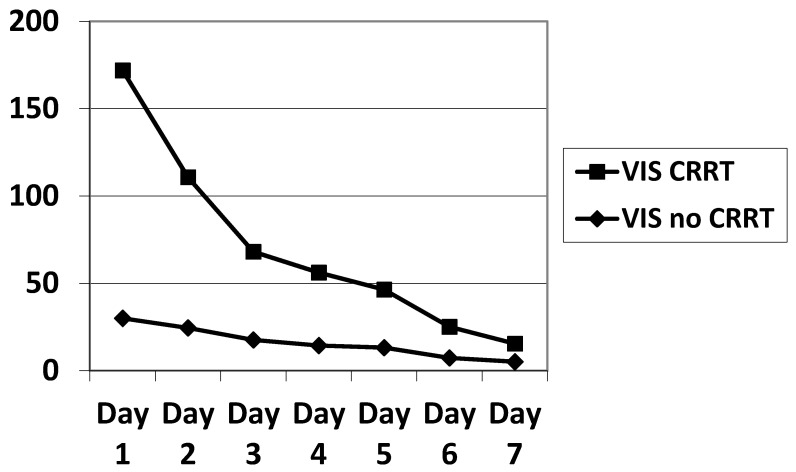
Evolution of the VIS during the 7 days of ECMO. VIS significantly elevated (*p* < 0.05) on day 1, 2, 3 in the CRRT population. VIS: vasoactive inotropic score; ECMO: extracorporeal membrane oxygenation.

In our small sample, there were slightly more males than females. The PIM, PRISM, PELOD, P-PREP and Lung Injury Score (LIS) scores were statistically equivalent. The VIS was significantly higher in ECMO patients on CRRT during the first three days of ECMO (Table 2, Figure 2), as was the total amount of noradrenalin administered (Table 1). We calculated the PELOD and P-PREP scores without the renal component, and found no significant difference. The ventilator parameters characterizing the severity of ARDS showed no significant difference (Table 3).

The “propensity score” on all these variables as mentioned above permitted us to obtain the same significant results except for the maximum creatinine (Table 4).

CRRT patients received more red blood cells (RBC), platelets (PLT) and fresh frozen plasma (PFC) during the 7 days of ECMO and during the CRRT period (Table 5).

**Figure 3:**
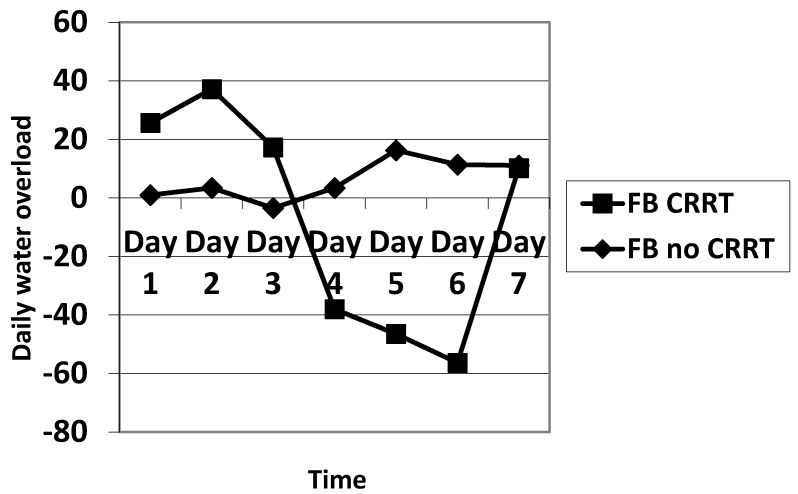
Comparison of the evolution of the fluid balance during the 7 days of ECMO between CRRT and no CRRT. FB: fluid balance.

**Figure 4:**
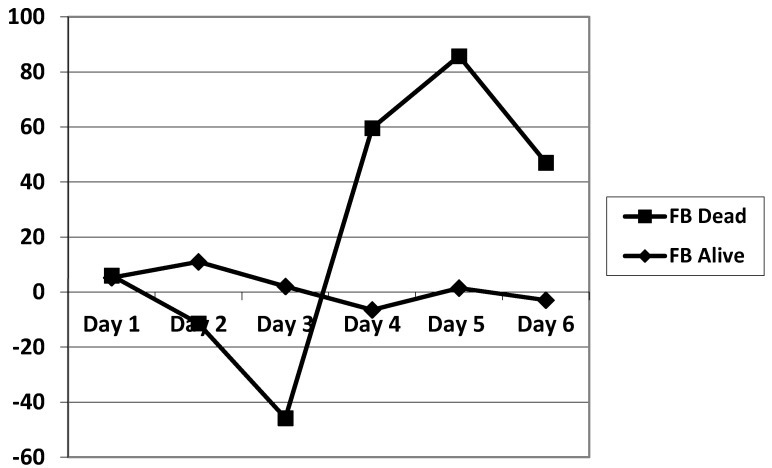
Comparison of the evolution of the fluid balance during the 7 days of ECMO between survivors and non-survivors. FB: fluid balance.

The daily fluid balance was significantly lower from day 4 to day 6 in the CRRT group. The cumulative diuretic administration was not significantly different between the two groups. The duration of ECMO and ICU hospitalization were identical between the two groups. There was no significant difference in mortality between the two groups. Cumulative fluid balance was found to be higher in CRRT patients during the first three days (*p* ranging from 0.11 to 0.29) and lower on day 5 to day 7 (*p* = 0.31 on day 5 and *p* = 0.16 on day 7) compared with non-CRRT patients; this did not achieve statistical significance. Of note, CRRT resulted in a cumulative fluid balance that remained negative from day 5 onward (Figure 2). Although daily fluid balance during the first 3 days of ECMO was more positive in the CRRT group, the CRRT group had a more negative balance from day 4 to day 7, as shown in Figure 3. We attempted to assess mortality based on the daily fluid balance, but could not find any association since the number of deceased patients was below 5 at day 2, and thus did not allow for statistical analysis (Figure 4). Nevertheless, non-survivors seem to have a more positive fluid balance from day 4.

Regarding mortality, we compared the population of survivors to the population of non-survivors (Table 6). We found a higher frequency of meconium inhalation in the survivor population (48% vs. 0%, *p* = 0.007) and a lower frequency of sepsis (5% vs. 40%, *p* = 0.01). The PIM score was higher in survivors than in non-survivors (10 [2.8–22.7] vs. 2.7 [0.8–7.6], *p* = 0.01). On the other hand, the P-PREP score was lower in the survivors (6 [2.0–8.0] vs. 10 [5.5–11], *p* = 0.02). We did not find any significant difference concerning the VIS score, the prevalence of CRRT, the duration of ECMO or the length of stay.

## 4. Discussion

### 4.1. Mortality

We found no difference between the two groups in terms of mortality at discharge from the PICU. However, seven patients died in the group who did not receive CRRT while only three patients died in the CRRT group. There were very few confounding factors, and the two groups were statistically identical with respect to demographics, etiology and severity of PARDS, and severity of illness scores. All patients with meconium inhalation survived. This is a population of patients in whom prognosis is better following the physiological fall of pulmonary hypertension on day 5. On the other hand, the incidence of sepsis was higher in deceased patients, which could partly explain the increased mortality. However, the VIS scores were equivalent, the severity of the shocks was identical, but the tolerance was probably different from one patient to another. The severity scores were higher on admission, but without having any repercussions on mortality, which for us provides an additional argument in favor of the benefit of ECMO in meconium inhalation which constituted 48% of survivors.

Several pediatric studies have shown increased hospital mortality in patients treated with ECMO + CRRT compared to patients treated with ECMO alone [8,25,26,27,28,29]. All of these studies used veno-arterial (VA) ECMO commenced after heart surgery for congenital heart disease. On the other hand, Lou et al. showed that CRRT did not increase mortality in children on ECMO (97.7% on VA-ECMO). They concluded that their findings differed from the other studies because of different types of patients, different study designs, and a large number of potential confounders [30]. Recently, it was reported that there is no increase in mortality associated with CRRT and VV-ECMO (42%) or VA-ECMO (58%) in adults [22]. However, another study found that CRRT initiated prior to commencement of ECMO resulted in a higher risk of mortality [20].

### 4.2. Fluid Balance and Renal Function

Daily fluid balance was significantly more positive in CRRT patients on the first day of ECMO placement and then became statistically more negative on day 4 and day 6. The indications for CRRT in our study were to treat fluid overload and maintain euvolemia.

Creatinine was significantly higher in the CRRT group. However, when creatinine clearance was calculated with the Pottel formula [31], this significance disappeared. There was no statistical difference in the PELOD and P-PREP scores without the renal component. None of our patients had chronic kidney disease. The introduction of ECMO required rapid adjustments of inotropes and vasopressors which can potentially result in ischemia and reperfusion injury [13]. Moreover, volume overload increases both the renal venous and medullary pressures and in turn decreases filtration resulting in a positive fluid balance which can exceed the kidney’s natural excretion capacity [32].

### 4.3. Consumption of Blood Products

We found a higher requirement of red blood cells, platelets and fresh frozen plasma in the group treated with CRRT during the 7 days of ECMO and during the CRRT period. This could be explained by the patient’s blood being exposed to two circuits and two filters at the same time. Intravascular coagulation [16] and hemolysis [13] were observed. The proportion of blood exposed to foreign circuits and membranes is greater in children than in adults. The median quantity of blood transfused per kg in the CRRT + ECMO group was 114 mL/kg, compared to 56.5 mL/kg in the Antonucci adult study [22].

### 4.4. Impact on Outcomes

Obtaining euvolemia via CRRT may potentially reduce the duration of ventilation in keeping with the restrictive fluid strategies recommended in the literature for the management of ARDS [30]. However, we did not observe a significant difference in the duration of mechanical ventilation between the two groups in our study. We did not observe a statistically longer duration of ECMO or ICU stay in CRRT patients.

### 4.5. Hemodynamic Instability

CRRT patients had a greater hemodynamic instability, characterized by a higher VIS (139 ± 251 vs. 45 ± 71, *p* = 0.042), and a higher cumulative noradrenaline use (0.812 ± 1.14 mg/kg vs. 0.185 ± 0.342 mg/kg, *p* = 0.0033). In a prospective observational study of 36 critically ill children treated with CRRT (a total of 161 CRRT connections), hypotension occurred in 49.7% of connections to the CRRT circuit [33]. In that study, 5.2 mL/kg fluid loading and a 12.5% increase in vasopressors were required in 38% of CRRT connections. One reason for this hypotension is that the CRRT circuit uses up to 5 to 10% of the total blood volume of the child [33]. Another cause of hemodynamic instability may be related to an alteration in the segmental kinetics of the left ventricle that can occur in the early hours of CRRT; this is described in adults and remains controversial [34]. A third potential cause is the exposure of blood to two artificial membranes resulting in the release of cytokines which can in turn induce vasoplegia [35]. A final etiology may be the release of hemoglobin and ATP following hemolysis resulting from shear stress generated by differences in flow velocity. The released hemoglobin can induce vasoplegia via a pro-inflammatory effect [36]. ATP released during hemolysis can activate P2 purinergic receptors on the vascular endothelium, resulting in the synthesis of potent vasodilators such as nitric oxide and prostaglandins [37].

### 4.6. Pediatric Specificities of Creatinine Measurements in AKI

Sixteen patients in the study were neonates. Neonates have lower baseline creatinine values than the pediatric population because of low muscle mass. Some patients may be defined as having AKI simply because their creatinine increases from 0.1 mg/dL to 0.2 md/dL. This challenges the relevance of using the KDIGO definition in children in the neonatal period. In addition, there were discrepancies between the creatinine level and the prescription of CRRT. The maximum creatinine in our study was 1.16 ± 0.66 mg/dL. This discrepancy can be explained in infants and young children by the phenomenon of creatinine dilution in the relatively large volume of the ECMO circuit and the ECMO + CVVH dual circuit [38].

Propensity score matching refers to the matching of individuals from the treatment and control groups with similar or similar propensity score values. This matching method attempts to match each treated individual with one or more untreated individuals whose observable characteristics are as similar as possible. The purpose of the match is to construct a control group comparable to the treated group in order to allow an unbiased estimate of the effect of treatment on treated individuals. By performing this operation, we confirmed the results obtained on the entire population by reducing the risk of selection bias.

### 4.7. Limitations

This study was retrospective and monocentric. It included 16 neonatal patients suffering from meconium inhalation, patients in whom the physiopathology was considered to be PARDS but whose prognosis was better following the physiological fall of pulmonary hypertension on day 5. The measurements of PO_2_ and PCO_2_ were calculated from different arterial, venous and capillary measures, which rendered all comparisons, and calculation of the PaO_2_/FiO_2_ ratio, impossible. The sample was limited to 45 patients. The study’s strength resides in the homogeneity of the pathologies and associated severity of illness scores, allowing us to focus on the impact of the degree of AKI. Similarly, the monocentric character ensured homogeneity of patient care.

## 5. Conclusions

We observed that CRRT initiation in patients with PARDS treated with ECMO can be done without increasing ECMO duration, PICU length of stay or mortality. Patients on CRRT have greater hemodynamic instability during the initiation of ECMO and during the first 3 days of treatment. This partly explains the greater renal fragility and the use of CRRT. On the other hand, the addition of CRRT is accompanied by a greater use of blood products. The observed potential benefit is a significant decrease in the daily fluid balance at day 4 in the ECMO + CRRT patient group. Prospective studies are necessary to demonstrate a possible overall survival benefit.

## Figures and Tables

**Table 1:** Characteristics of the population studied.

	All (*n* = 45)	No CRRT (*n* = 37)	CRRT (*n* = 8)	*p*-Value
Age, months	3.5 [0.0–18.2]	1 [0.0–16.5]	6.5 [0.7–46.2]	0.3
Weight, kg	4.7 [3.6–9.8]	4.7 [3.6–8.7]	5.8 [3.5–16.2]	0.63
Male, *n* (%)	23 (51)	21 (56)	5 (62.5)	0.45
Pneumonia, *n* (%)	22 (48)	21 (56)	5 (62.5)	0.9
Sepsis, *n* (%)	6 (13)	5 (13)	1 (12)	0.98
Inhalation, *n* (%)	17 (37)	15 (42)	2 (25)	0.69
Scores				
PIM	8.0 [1.3–15.8]	8.0 [2.3–15.8]	2.9 [0.7–28.7]	0.25
PRISM	22.6 ± 13.1	22.6 ± 12.8	22.2 ± 15.7	0.71
PRISM Predicted mortality (%)	55.2 [18–88.9]	59.6 [20–88.5]	17.6 [4.1–98.4]	0.69
PELOD	10 [6.0–12.5]	9.5 [5.7–11.7]	11 [10–18]	0.15
PELOD (non- renal)	10 [6.0–12.5]	9.5 [5.7–11.7]	11 [10–18]	0.23
PELOD Predicted mortality (%)	10.7 [1.4–19.2]	8.5 [1.4–19.2]	14.5 [3.9–27.5]	0.24
P-PREP	6.0 [4.0–10]	7 [4.5–10]	5.5 [3.2–9.5]	0.87
P-PREP (non- renal)	0.5 [−2–2.7]	0.5 [−1.75–2.0]	1 [−2–3.75]	0.8
LIS	3.3 [3.0–3.7]	3.3 [3–3.7]	3.2 [2.6–3.6]	0.7
VIS max	30 [7.7–60.5]	21.6 [6.9–54.6]	44.5 [29.2–94.5]	0.042
Creatinine max mg/dl	0.7 ± 0.4	0.6 ± 0.3	1.1 ± 0.6	0.0032
Clearance min, ml/min	39 [25–63]	46 [27–66]	28 [21–31]	0.11
Fluid balance max, ml/kg	139 [35–264]	133 [20–264]	155 [103–345]	0.35
Lactate max, mg/dl	3.46 [2.0–6.0]	3.34 [1.9–6.0]	4.2 [2.5–6.8]	0.24
Pre-ECMO pH	7.16 [7.09–7.30]	7.17 [7.10–7.30]	7.13 [7.06–7.39]	0.62
Treatment				
NA, mg/kg	0.0 [0.02–0.28]	0.0 [0.0–0.21]	0.43 [0.0–1.0]	0.0033
Furosemide, mg/kg	21.7 [4.9–56.2]	20.3 [4.0–56.2]	39.0 [7.2–92.6]	0.33
NO, *n* (%)	33 (73)	27 (72.9)	6 (75)	0.83
Corticoids, *n* (%)	12 (27)	8 (21)	4 (50)	0.17
Ig, *n* (%)	5 (11)	5 (13)	0 (0)	0.56
NMBA, *n* (%)	40 (88)	33 (94)	7 (87)	0.98
ECMO, duration, h	126 [92–177]	121 [90–175]	159 [111–271]	0.15
ICU LOS, d	15 [9.7–26.0]	13.5 [9.7–20.2]	24.0 [10.7–71.0]	0.16
ICU mortality, *n* (%)	9 (20)	7 (18)	3 (37)	0.71

CRRT: continuous renal replacement therapy; PIM: Pediatric Index Mortality; PRISM: Pediatric Risk Mortality; PELOD: Pediatric Logistic Organ Dysfunction; P-PREP: Pediatric Pulmonary Rescue with Extracorporeal Membrane Oxygenation Prediction; LIS: Lung Injury Score; VIS: Vasoactive Inotropic Score; Max: maximum; Clear: clearance; Min: minimum; ECMO: Extra Corporeal Membrane Oxygenation; NA: noradrenaline; Ig: immune globulin; NO: Nitric oxide, NMBA: Neuromuscular blocking agents; ICU: intensive care unit; LOS: length of stay.

**Table 2:** Evolution of the VIS as a function of time showing hemodynamic instability in the period of ECMO preceding the initiation of CRRT.

	VIS no CRRT	VIS CRRT	*p*
Day 1	20 [0–117]	65 [14–379]	0.0003
Day 2	10 [0–107]	92.5 [7–167]	0.0035
Day 3	7.2 [0–93]	37 [0–107]	0.0231
Day 4	10 [0–75]	20 [3–104]	0.0671
Day 5	4 [0–87]	15 [0–102]	0.0898
Day 6	0 [0–116]	7.5 [0–63]	0.0477
Day 7	0 [0–76]	5 [0–33]	0.1284

VIS: vasoactive inotropic score; CRRT: continuous renal replacement therapy; ECMO: extracorporeal membrane oxygenation.

**Table 3:** The ventilator parameters characterizing the severity of ARDS showed no significant difference.

	No CRRT	CRRT	*p*
OSI	11.1 [3.2–26.3]	17.4 [7.8–24.3]	0.0663
Tidal Volume, mL/kg	3.7 [1–14]	4.9 [1.8–8.7]	0.6706
PEEP, cm H2O	8.5 [2–16]	9 [5–12]	0.9551
Plateau, cm H2O	25.5 [15–40]	30 [24–32]	0.0721
Driving pressure, cm H2O	16 [4–30]	17.5 [0–25]	0.5742
P/V, cm H2O/L	6.75 [2–18]	6 [3.4–13.3]	0.9921
Compliance mL/cm H2O	1.12 [0.3–31.8]	2.1 [0.2–4.5]	0.7915

OSI: oxygen saturation index; PEEP: positive end-expiratory pressure; P/V: pressure on volume per kilogram.

**Table 4:** Statistics after propensity score 2:1.

	Control (*n* = 16)	CRRT (*n* = 8)	*p*-Value
Age, months	0.0 [0.0–16.5]	6.5 [0.7–46.2]	0.21
Weight, kg	3.9 [3.4–10.0]	5.8 [3.5–16.2]	0.44
Male, *n* (%)	9 (56)	5 (62.5)	0.66
Pneumonia, *n* (%)	5 (31)	5 (62.5)	0.65
Sepsis, *n* (%)	3 (18)	1 (12)	>0.99
Inhalation, *n* (%)	7 (43)	2 (25)	0.65
Scores			
PIM	12.8 [3.8–32.4]	2.9 [0.7–28.7]	0.13
PRISM	27.0 ± 10.5	22.2 ± 15.7	0.4
PRISM Predicted mortality (%)	45.2 ± 33.7	36.4 ± 43.5	0.61
PELOD	10.5 [7.5–20.0]	11 [10–18]	0.15
PELOD Predicted mortality (%)	14.5 [4.7–19.2]	14.5 [3.9–27.5]	0.61
P-PREP	7.8 ± 2.9	5.7 ± 4.3	0.17
P-PREP (non- renal)	0.6 ± 2.8	0.75 ± 2.8	0.95
LIS	3.4 [3.0–3.9]	3.2 [2.6–3.6]	0.55
VIS max	16.3 [6.7–34.0]	44.5 [29.2–94.5]	0.01
Creatinine max mg/dl	0.8 ± 0.3	1.1 ± 0.6	0.17
Clearance min, ml/min	32 [18-64]	28 [21–31]	0.7
Fluid balance max, ml/kg	71 [12–258]	155 [103–345]	0.23
Lactate max, mg/dl	4.0 [2.7–6.0]	4.2 [2.5–6.8]	0.78
Pre-ECMO pH	7.18 ± 0.15	7.18 ± 0.19	0.79
Treatment			
NA, mg/kg	0.0 [0.0–0.17]	0.43 [0.0–1.0]	0.005
Furosemide, mg/kg	26.5 [5.2–44.2]	39.0 [7.2–92.6]	0.37
ECMO, duration, h	105 [−302]	159 [111–271]	0.37
ICU LOS, d	15 [10.0–33.2]	24.0 [10.7–71.0]	0.42
ICU mortality, *n* (%)	4 (25)	3 (37)	0.64

CRRT: continuous renal replacement therapy; PIM: Pediatric Index Mortality; PRISM: Pediatric Risk Mortality; PELOD: Pediatric Logistic Organ Dysfunction; P-PREP: Pediatric Pulmonary Rescue with Extracorporeal Membrane Oxygenation Prediction; LIS: Lung Injury Score; VIS: Vasoactive Inotropic Score; Max: maximum; Clear: clearance; Min: minimum; ECMO: Extra Corporeal Membrane Oxygenation; NA: noradrenaline; ICU: intensive care unit; LOS: length of stay.

**Table 5:** Marked effect of CRRT on blood derivative consumption.

	No CRRT	CRRT	*p*
RBC, mL/kg	39 [26–62]	114 [76–174]	0.0005
RBC, mL/kg after CRRT	35 [17–54]	74 [45–160]	0.015
PLT, mL/kg	20 [8–43]	83 [68–152]	0.0002
PLT mL/kg after CRRT	13 [0–36]	55 [16–145]	0.0014
FFP, mL/kg	0 [0–10]	36 [6–53]	0.0025
FFP, mL/kg after CRRT	0 [0–0.8]	17 [0–35]	0.0303

CRRT: Continuous renal replacement therapy; RBC: Red blood cells; PLT: Platelets; FFP: Fresh frozen plasma.

**Table 6:** Comparison of survivors vs. non survivors.

	All (*n* = 45)	Survivors (*n* = 35)	Non-Survivors (*n* = 10)	*p*-Value
Age, months	3.5 [0.0–18.2]	2 [0.0–18.7]	4.5 [0.7–46.2]	0.56
Weight, kg	4.7 [3.6–9.8]	4.2 [3.5–9.4]	5.0 [3.9–10.8]	0.38
Male, *n* (%)	26 (57)	20 (57)	6 (60)	0.72
Pneumonia, *n* (%)	22 (50)	16 (55)	6 (60)	0.48
Sepsis, *n* (%)	6 (13)	2 (5)	4 (40)	0.01
Inhalation, *n* (%)	17 (37)	17 (48)	0 (0)	0.007
Scores				
PIM	8.0 [1.3–15.8]	10 [2.8–22.7]	2.7 [0.8–7.6]	0.01
PRISM	22.6 ± 13.1	23.9 ± 13.9	17.3 ± 8.4	0.18
PRISM Predicted mortality (%)	55.2 [18–88.9]	58.0 [19–91.1]	17.8 [6.9–75.3]	0.16
PELOD	10 [6.0–12.5]	9.5 [6.0–11.0]	13 [8.7–20]	0.06
PELOD (non- renal)	10 [6.0–12.5]	9.5 [6.0–11.0]	13 [8.7–20]	0.06
PELOD Predicted mortality (%)	10.7 [1.4–19.2]	8.5 [1.3–18.4]	16.2 [10–32]	0.06
P-PREP	6.0 [4.0–10]	6 [2.0–8.0]	10 [5.5–11]	0.02
P-PREP (non- renal)	0.5 [−2–2.7]	−1 [−2.0–2.0]	3 [2.0–4.0]	<0.0001
LIS	3.3 [3.0–3.7]	3.3 [3.0–3.7]	3.1 [2.3–3.7]	0.32
VIS max	30 [7.7–60.5]	30 [10–79]	29 [5.0–57]	0.62
Creatinine max mg/dl	0.7 ± 0.4	0.75 ± 0.36	0.76 ±0.65	0.52
Clearance min, ml/min	39 [25–63]	39 [26–62]	43 [21–71]	0.91
Fluid balance max, ml/kg	139 [35–264]	133 [28–244]	139 [36–377]	0.58
Lactate max, mg/dl	3.46 [2.0–6.0]	4.0 [2.0–6.2]	2.3 [1.6–3.1]	0.03
Pre-ECMO pH	7.16 [7.09–7.30]	7.16 [7.10–7.30]	7.20 [7.07–7.28]	0.62
Treatment				
CVVH, *n* (%)	8 (17)	5 (11)	3 (30)	0.34
NA, mg/kg	0.02 [0.00–0.28]	0.01 [0.0–0.21]	0.22 [0.0–0.8]	0.47
Furosemide, mg/kg	21.7 [4.9–56.2]	21.2 [5.0–58.0]	18.8 [1.8–57.46]	0.64
ECMO, duration, h	126 [92–177]	126 [99–177]	107 [41–232]	0.46
ICU LOS, d	15 [9.7–26.0]	15 [9.2–27.5]	16 [10.0–27.2]	0.81

CRRT: continuous renal replacement therapy; PIM: Pediatric Index Mortality; PRISM: Pediatric Risk Mortality; PELOD: Pediatric Logistic Organ Dysfunction; P-PREP: Pediatric Pulmonary Rescue with Extracorporeal Membrane Oxygenation Prediction; LIS: Lung Injury Score; VIS: Vasoactive Inotropic Score; Max: maximum; Clear: clearance; Min: minimum; ECMO: Extra Corporeal Membrane Oxygenation; NA: noradrenaline; ICU: intensive care unit; LOS: length of stay.

## Data Availability

Not applicable.
